# Techniques to Detect and Quantify the Bacterial Metalloprotease AprX in Bovine Milk: A Review

**DOI:** 10.1111/1541-4337.70415

**Published:** 2026-02-08

**Authors:** Aritra Sinha, Alan L. Kelly

**Affiliations:** ^1^ School of Food and Nutritional Sciences University College Cork Cork Ireland; ^2^ Dairy Processing Technology Centre University College Cork Cork Ireland

## Abstract

The heat‐stable metalloprotease AprX, secreted by psychrotrophic *Pseudomonas* spp., is a major cause of quality deterioration in dairy products, particularly ultrahigh temperature (UHT) milk. This review synthesizes the evolution and current state of detection and quantification techniques for AprX in bovine milk, covering traditional immunological assays, enzymatic activity measurements, and zymography, alongside modern molecular approaches such as PCR‐based methods, mass spectrometry, and advanced biosensors. Highly sensitive systems, including indirect ELISA (LOD 21.0 ng/mL), multiplex qPCR, and biosensor platforms, have enhanced the ability to detect AprX activity at low concentrations. Comparative analysis evaluates these methods in terms of sensitivity, specificity, turnaround time, cost, ease of use, and industrial applicability. Emerging directions such as multiomics integration, biosensor miniaturization, and artificial intelligence‐driven data interpretation are also discussed. By critically assessing available and emerging tools, this review supports dairy scientists and industry stakeholders in selecting optimal strategies to detect, monitor, and mitigate AprX‐associated spoilage in milk and dairy products.

## 1 Introduction

The dairy industry faces significant challenges in maintaining product quality and shelf life, particularly for ultrahigh temperature (UHT)‐treated milk and milk products. Among these challenges, the presence of heat‐stable proteases in raw milk represents a major concern due to their ability to remain active even after severe thermal processing (Datta and Deeth 2003; C. Zhang et al. 2019). An alkaline metalloprotease, AprX, secreted by psychrotrophic bacteria of the genus *Pseudomonas*, has emerged as one of the most problematic of these enzymes, capable of causing quality defects such as bitter flavors, gelation, and protein destabilization in processed milk products during storage (Baglinière et al. 2013; Stoeckel et al. 2016; Matéos et al. 2015; Andreani et al. 2016).

Psychrotrophic bacteria, particularly *Pseudomonas* species, can grow at refrigeration temperatures (4°C–7°C) and often dominate the microbiota of cold‐stored raw milk prior to processing (Sørhaug and Stepaniak 1997; Lafarge et al. 2004; De Jonghe et al. 2011; Decimo et al. 2014; Quigley et al. 2013; Yap et al. 2024). While the bacteria themselves are readily inactivated by pasteurization and UHT treatments (Guo et al. 2024; Narvhus et al. 2021; Xu et al. 2024), the extracellular proteases they produce during cold storage can exhibit remarkable heat stability (Machado et al. 2017; Aguilera‐Toro et al. 2023a). Studies have demonstrated that AprX proteases can retain 25%–88% of their activity even after UHT treatment (138°C for 18–20 s), allowing them to continue destabilizing milk proteins throughout the product's shelf life (Glück et al. 2016, 2021; Vithanage et al. 2016)

The prevalence of *Pseudomonas* species in raw milk has been extensively documented. Quigley et al. (2013) reported that *Pseudomonas* can constitute up to 70%–90% of the psychrotrophic population in refrigerated raw milk. von Neubeck et al. (2015, 2017) characterized the biodiversity of refrigerated raw milk microbiota and found that *Pseudomonas* was the predominant genus, with *Pseudomonas proteolytica*, *Pseudomonas gessardii*, *Pseudomonas fluorescens*, and *Pseudomonas fragi* being particularly common. More recently, Maier et al. (2020) identified different AprX–lipA2 operon structures among *Pseudomonas* species isolated from raw milk and established a correlation between operon organization and proteolytic potential. It was concluded that the genetic structure of the *aprX–lipA* operon affects the spoilage potential of *Pseudomonas* species. However, this influence is strain specific and results from a complex interplay between intrinsic bacterial traits and external environmental factors.

Environmental factors known to influence AprX production include temperature (S. Zhang et al. 2015), medium composition (C. Zhang et al. 2019), the presence of calcium (Martins et al. 2015), and iron content (Woods et al. 2001). The combined effects of these environmental conditions and genetic factors result in highly heterogeneous proteolytic activity among *Pseudomonas* species in UHT milk. Recent work has highlighted that AprX is not a single uniform enzyme type in milk, with different AprX endopeptidases showing distinct heat stability and substrate selectivity (Volk et al. 2025). Another recent peptidomic evaluation suggests that low‐temperature inactivation may have limited effectiveness in reducing AprX‐mediated hydrolysis in UHT milk, reinforcing the need for prevention and sensitive detection (C. Zhang et al. 2023). Given this complexity, accurate methods for predicting the spoilage potential of these bacteria are still lacking in the dairy industry (Aguilera‐Toro et al. 2023b; Maier et al. 2021; Marchand et al. 2009b).

The economic impact of AprX‐related spoilage is substantial, with premature product deterioration leading to consumer complaints, product recalls, and financial losses for dairy processors (Datta and Deeth 2003; Stoeckel et al. 2016). Baur et al. (2015) estimated that proteolytic spoilage accounts for approximately 5%–10% of dairy product waste globally. In addition, the increasing global trend toward extended shelf‐life dairy products amplifies the importance of controlling and detecting these heat‐resistant enzymes (C. Zhang et al. 2019). As a result, there has been growing interest in developing effective methods to detect and quantify AprX in milk, both for dairy research and as quality control tools for the dairy industry.

The detection and quantification of AprX in milk present several technical challenges. First, milk is a complex biological fluid containing numerous proteins, lipids, and other components that can interfere with detection methods (Volk et al. 2021). Second, AprX may be present at very low concentrations while still being capable of causing spoilage over time (Matéos et al. 2015). Third, there are multiple *Pseudomonas* species that produce variants of AprX with different properties and activities (Marchand et al. 2009a; Maier et al. 2020). These challenges have driven the development of increasingly sophisticated detection techniques over the past four decades.

The AprX gene, which encodes the AprX metalloprotease, is part of the AprX–lipA2 operon found in many *Pseudomonas* species (Woods et al. 2001). This operon typically includes genes encoding the metalloprotease (AprX), its inhibitor (aprI), and genes involved in secretion (*aprD*, *aprE*, *aprF*) (Maier et al. 2020; Martins et al. 2005). The AprX enzyme belongs to the metzincin superfamily of metalloproteases, characterized by a zinc‐binding motif in the active site (Rawlings et al. 2014). The molecular weight of AprX typically ranges from 45 to 50 kDa, though smaller proteolytic fragments have been observed in some studies (Stuknytė et al. 2016).

The proteolytic activity of AprX primarily targets caseins in milk, with particular affinity for β‐casein and κ‐casein (Matéos et al. 2015; Baglinière et al. 2013, Stuknytė et al. 2016). This proteolysis can lead to destabilization of casein micelles, resulting in age gelation, sedimentation, and bitter flavor development in UHT milk during storage (C. Zhang et al. 2019). The rate and extent of proteolysis depend on several factors, including the specific *Pseudomonas* species, storage conditions, and initial bacterial load (Nicodeme et al. 2005; Caldera et al. 2016).

**FIGURE 1:**
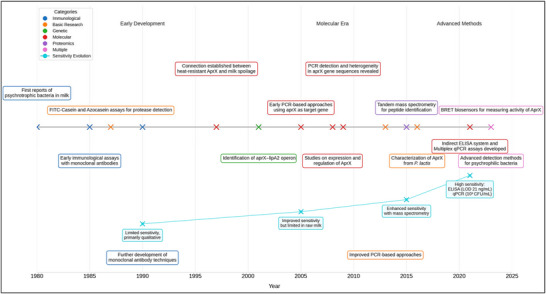
Chronological evaluation of AprX detection methods in bovine milk.

This review provides a comprehensive overview of the techniques used to detect and quantify metalloprotease AprX in bovine milk, from early immunological methods to cutting‐edge molecular approaches. Additionally, the chronological development of these techniques (Figure 1), their respective strengths and limitations, and emerging trends in AprX detection were traced, analyzed, and explored, respectively. By synthesizing this information, we aim to provide dairy scientists, quality control professionals, and researchers with valuable insights into the current state of AprX detection and future directions in this field.

### 1.1 Literature Selection and Scope

This review focuses on analytical methods that enable the detection, quantification, or functional interpretation of the heat‐stable metalloprotease AprX in milk and dairy‐relevant matrices. Studies were included if they (i) directly targeted AprX at the protein, activity, or gene level or (ii) provided methodologically transferable approaches applicable to AprX detection in dairy systems.

## 2 Methods for AprX Detection and Quantification

### 2.1 ELISA Systems

Earlier immunological approaches to detecting *Pseudomonas* AprX proteases (Birkeland et al. 1985; Clements et al. 1990; Matta et al. 1997) primarily relied on antibodies directed against strain‐specific proteases. Enzyme‐linked immunosorbent assay (ELISA) is a highly specific immunological method for detecting the heat‐stable protease AprX in milk. For detection, the *aprX* antigen is bound to a solid‐phase microplate (e.g., polystyrene) that selectively adsorbs the enzyme and corresponding antibodies while minimizing nonspecific binding. An enzyme label—commonly alkaline phosphatase or peroxidase—conjugated to the detection antibody catalyzes a substrate reaction, producing a measurable yellow (alkaline phosphatase) or brown (peroxidase) color within 30–60 min. The reaction is stopped with acid or alkali, and the absorbance is measured at the appropriate wavelength (typically 400–600 nm) to quantify AprX levels (Aydin 2015). In direct ELISA formats, the enzyme‐labeled primary antibody binds directly to *aprX*, offering a shorter protocol but increasing the risk of false positives, as it cannot distinguish between active and denatured/inactive enzyme.

In an indirect ELISA, *aprX* bound to the solid phase is first detected by a primary antibody, which is then recognized by an enzyme‐linked secondary antibody that produces the measurable signal. Volk et al. (2021) developed an indirect ELISA specifically designed to detect *Pseudomonas aprX*, incorporating a two‐step sample preparation protocol to mitigate milk protein interference, an advancement not reported in earlier routine direct ELISA assays. Initially, trisodium citrate destabilizes casein micelles by chelating Ca^2+^, followed by a 10‐fold concentration of AprX via hydrophobic interaction chromatography.

**FIGURE 2:**
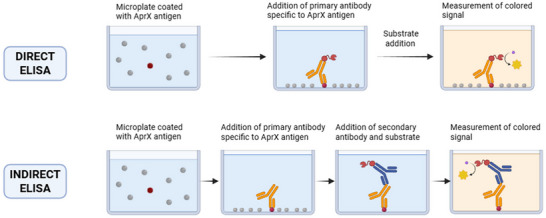
Comparative overview of direct and indirect enzyme‐linked immunosorbent assay (ELISA) methods for AprX detection.

The indirect ELISA process involves coating a microtiter plate with the prepared milk sample, adding specific anti‐*aprX* antibodies (produced against purified AprX from *Pseudomonas lactis*), and detecting antibody binding with a secondary enzyme‐conjugated antibody, followed by a colorimetric substrate reaction (Volk et al. 2021). This method achieved a limit of detection (LOD) of 21.0 ng/mL, a limit of quantification (LOQ) of 25.7 ng/mL, and high precision (intraday CV: 0.2%–0.8%; interday CV: 5.6%–6.8%). Recovery rates ranged from 92.3 ± 1.6% to 105 ± 4.7%, with a minimum quantifiable AprX activity of 500 pkat (1 picokatal = 10^−12^ moles of substrate converted per second) Na‐caseinate/OPA per milliliter in spiked milk. Western blot analysis confirmed that antibody specificity, dependent on *aprX* sequence homology across *Pseudomonas* species, ensures high selectivity but may limit detection for divergent strains (Volk et al. 2021). A comparative overview of the direct and indirect methods is shown in Figure 2.

Birkeland et al. (1985) developed a sandwich ELISA using polyclonal antibodies raised in rabbits against purified *P. fluorescens* P1 proteinase, achieving high sensitivity with a detection limit of 0.25 ng/mL in milk and cream. However, the assay's specificity was restricted to proteinases immunologically related to the P1 strain, limiting its effectiveness for broader detection across diverse *Pseudomonas* strains.

Clements et al. (1990) introduced an inhibition ELISA for detecting heat‐stable proteases from four *P. fluorescens* strains in UHT milk, with detection limits ranging from 0.24 to 7.8 ng/mL and a total assay time of 6 h. This method also required homologous antigen–antibody pairs for each protease, reducing its applicability due to the known immunological heterogeneity among *Pseudomonas* proteases. Later, Matta et al. (1997) developed a dot‐ELISA using IgG antibodies against the AFT‐36 protease, achieving a detection limit of 1.01 ng/mL in buffer and milk within 2.5 h under ambient conditions. Despite its simplicity and portability, the assay was limited by strain‐specific recognition and failed to detect proteases from certain *Pseudomonas* species. A comparative overview of all ELISA methods to detect AprX is shown in Table 1.

**TABLE 1:** Comparative overview of ELISA‐based methods for AprX detection.

Type	Principle	Advantages	Disadvantages	Author	Comments
Direct ELISA (general)	Enzyme‐labeled primary antibody binds directly to AprX	Shorter protocol; simpler procedure; fewer steps	Higher false positive risk; cannot distinguish active versus denatured enzyme; less specific than indirect methods	Various	Basic format, less commonly used for AprX
Indirect ELISA with sample prep	Two‐step sample preparation: citrate treatment + hydrophobic interaction chromatography; anti‐AprX antibodies from *P. lactis*	Excellent LOD (21.0 ng/mL) and LOQ (25.7 ng/mL); high precision (CV: 0.2%–0.8% intraday); good recovery (92.3%–105%); mitigates milk protein interference; high selectivity	Complex sample preparation; may miss divergent strains; depends on sequence homology; cannot distinguish active versus inactive enzyme	Volk et al. 2021	Most advanced ELISA protocol with specialized sample preparation
Sandwich ELISA	Polyclonal antibodies from rabbits against purified *P. fluorescens* P1 proteinase	High sensitivity (LOD: 0.25 ng/mL); works in milk and cream	Strain‐specific (P1 only); limited cross‐strain detection; poor effectiveness for diverse *Pseudomonas* strains	Birkeland et al. 1985	First reported ELISA for *Pseudomonas* proteases
Inhibition ELISA	Detection of heat‐stable proteases from four *P. fluorescens* strains in UHT milk	Multiple strain detection (four strains); good sensitivity (LOD: 0.24–7.8 ng/mL)	Long assay time (6 h); requires homologous antigen–antibody pairs for each protease; limited by immunological heterogeneity	Clements et al. 1990	Specifically designed for UHT milk analysis
Dot‐ELISA	IgG antibodies against AFT‐36 protease	Simple and portable; fast (2.5 h); ambient temperature operation; LOD: 1.01 ng/mL	Strain‐specific recognition; failed to detect certain *Pseudomonas* species; limited broad applicability	Matta et al. 1997	Field‐deployable format

A general limitation of ELISA‐based methods is that they primarily detect the presence of the protease protein or, in nucleic acid–based formats, its corresponding gene rather than its actual enzymatic activity. As a result, they cannot discriminate between catalytically active, partially denatured, or completely inactive enzyme molecules. Any residual, misfolded, or heat‐inactivated AprX, for example, will still be recognized by the antibody as long as the epitope is preserved. This can lead to false positives and an overestimation of spoilage risk, since the assay reports immunoreactivity rather than the real proteolytic potential in the sample.

### 2.2 Activity‐Based Assays

#### 2.2.1 Azocasein Assay

The azocasein assay is one of the earliest and most widely used methods for evaluating proteolytic activity in dairy systems, with its application dating back to the 1980s (Christen and Marshall 1984). The substrate, azocasein, consists of casein chemically coupled to an azo dye. When AprX hydrolyzes the peptide bonds within azocasein, smaller, soluble peptide fragments containing the dye are released. Unhydrolyzed azocasein and larger fragments are then precipitated, typically using trichloroacetic acid (TCA), and the absorbance of the colored supernatant, measured at a specific wavelength (e.g., 366 or 440 nm, depending on the dye), is proportional to the amount of substrate cleaved and thus to the protease activity. One unit of protease activity is typically defined as the amount of enzyme required to hydrolyze azocasein to give an increase of 1 unit of absorbance per milliliter of sample per minute (Paludetti et al. 2020).

The azocasein assay provides a quantitative measure of protease activity and has been successfully applied to assess proteolytic activity in milk samples inoculated with *Pseudomonas* and even in other dairy products like Ricotta (Andreani et al. 2016). Researchers have used this assay to classify *Pseudomonas* strains based on their proteolytic activity levels. For example, strains can be categorized as “high producers” (extracellular protease activity >500 Δ*A*/h·mL), “moderate producers,” or “low producers” based on their azocasein assay results (Maier et al. 2020).

Such a method is relatively straightforward and uses readily available reagents. However, its sensitivity is often considered insufficient for detecting low levels of activity relevant to long‐term UHT spoilage. A major limitation is its lack of specificity; it measures the activity of all proteases capable of hydrolyzing azocasein under the assay conditions (Dacres et al. 2023). In milk, this includes the native protease plasmin, making it difficult to attribute the measured activity solely to AprX. Potential interference from sample turbidity or color can also occur if sample preparation is inadequate, while the requirement for a fixed incubation time limits its use for rapid analysis.

Furthermore, no standardized protocol exists, and different authors have applied varying incubation times, wavelengths, and activity definitions, making cross‐study comparisons difficult (Aguilera‐Toro et al. 2023b; Dufour et al. 2008; Maier et al. 2020; Martins et al. 2015). Despite these limitations, its use persists in research, likely due to its simplicity and cost‐effectiveness when absolute specificity is not the primary requirement or when comparing relative activities under defined conditions (Ribeiro Júnior et al. 2018)

#### 2.2.2 Trinitrobenzenesulfonic Acid (TNBS) Assay

This method quantifies the extent of proteolysis by measuring the generation of new amino groups. Proteolytic cleavage of peptide bonds exposes new N‐terminal α‐amino groups. The TNBS reagent reacts specifically with these primary amines under alkaline conditions (pH ∼9.2) in the dark, forming a yellow‐orange derivative (trinitrophenyl‐amine). The intensity of this color, measured spectrophotometrically (around 420 nm), is proportional to the concentration of free amino groups released (Andreani et al. 2016). The increase in absorbance over time or compared to an initial time point or control sample reflects the extent of protein hydrolysis. Results are usually expressed as millimoles of free amino groups per gram of protein, using a calibration curve with L‐lysine or glycine.

The TNBS assay provides a quantitative measure of the total peptide bond cleavage that has occurred in a sample. It has been used to assess the overall proteolytic spoilage in stored milk samples (Meng et al. 2017). Like the azocasein assay, it is nonspecific and detects the cumulative action of all active proteases (including AprX and plasmin) in the sample and measures the extent of hydrolysis rather than the initial rate of reaction. The assay requires incubation time both for the proteolysis to occur in the sample and for the color development reaction with TNBS. Its sensitivity for detecting subtle changes early in storage might be limited, and results can be influenced by the baseline level of free amino acids and small peptides already present in the milk sample (Andreani et al. 2016).

#### 2.2.3 Zymography

Casein zymography has been employed as a visual technique to detect and characterize AprX proteases. Stuknytė et al. (2016) used this approach to study the extracellular thermostable proteolytic activity of *P. fluorescens PS19* on bovine caseins. Their zymogram revealed a thermostable protease of approximately 45 kDa, which was assigned to AprX metalloprotease secreted by *P. fluorescens*. After concentration by ultrafiltration at 10 kDa, the heat‐treated cell‐free supernatant showed two additional thermostable proteolytic bands on the zymogram, with molecular masses of approximately 15 and 25 kDa.

While zymography provides visual confirmation of proteolytic activity and can detect multiple proteases simultaneously, it is generally considered semiquantitative and requires extensive post‐run processing (renaturation, incubation in development buffer, staining, and destaining), making it more time‐consuming than other methods. Signal intensity depends on gel conditions, substrate loading, diffusion, and incubation time, so band density only approximates relative activity. In addition, co‐migration of proteins can complicate interpretation, and low‐abundance enzymes may fall below the detection limit. Despite these limitations, it remains a valuable tool for characterizing proteolytic enzymes, assessing their molecular weight, and confirming their activity specifically against milk proteins within a complex mixture.

#### 2.2.4 Milk Agar Plate Assay (Nonspecific Preliminary Screening Tool)

This is a basic microbiological screening method where bacteria are cultured on an agar medium containing a milk component, typically skim milk powder. Proteolytic organisms, such as many *Pseudomonas* strains, secrete extracellular proteases (like AprX) that diffuse into the agar and hydrolyze the opaque casein proteins. This enzymatic breakdown creates a visible zone of clearing or transparency (a halo) around the bacterial colony (Zarei et al. 2020).

This method is primarily qualitative, indicating whether a colony possesses caseinolytic activity. It can be considered semiquantitative if the diameter of the clearing zone is measured, although this correlates variably with actual enzyme production levels. Its main advantages are simplicity, low cost, and the direct visual link between a specific colony and proteolytic capability. However, it is slow, requiring time for bacterial growth and enzyme diffusion (days, especially at low temperatures). It is also nonspecific, detecting any organism producing casein‐breakdown enzymes, not just AprX‐producing *Pseudomonas*, and its sensitivity depends on the level of enzyme production and the incubation conditions. It serves primarily as a preliminary screening tool in microbiological surveys (Ribeiro Júnior et al. 2018) and is included in this review solely to provide historical and practical context as a preliminary screening tool, and not as a definitive approach for AprX detection or quantification. However, skim milk agar plates are best used as an initial flag for proteolytic potential, with colonies of interest subsequently subjected to more specific confirmatory methods such as AprX‐targeted ELISA, polymerase chain reaction (PCR)/quantitative PCR (qPCR), or peptidomic profiling to verify their spoilage relevance in dairy systems.

#### 2.2.5 Fluorescein Isothiocyanate (FITC)‐Casein Assay

**FIGURE 3:**
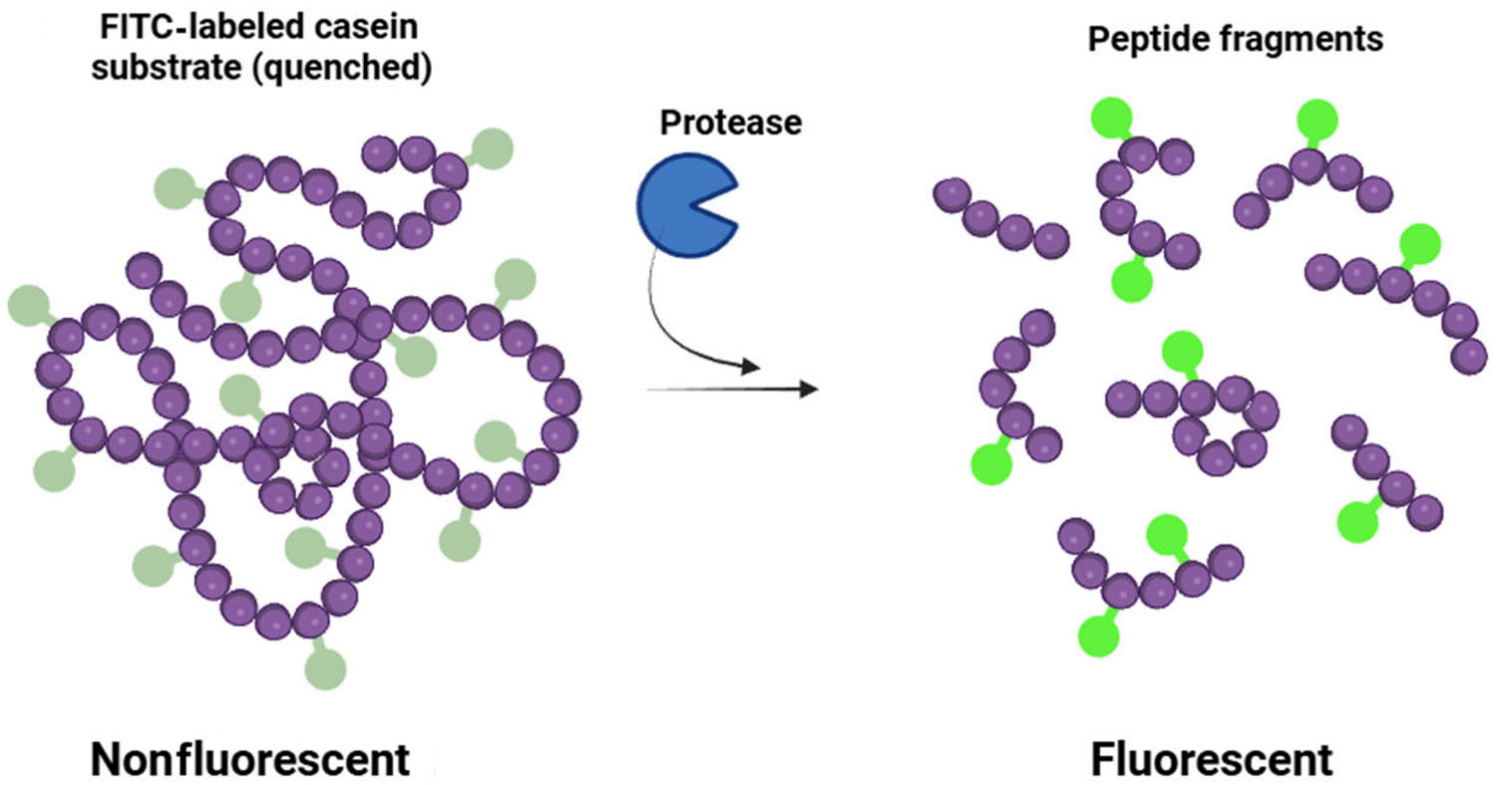
Schematic representation of the FITC casein assay principle.

This method, like the azocasein assay, originates from the 1980s (Christen and Senica 1987) and involves casein covalently labeled with a fluorescent dye, typically FITC. Protease activity cleaves the FITC‐casein, releasing smaller, soluble fluorescent peptides into the supernatant after precipitation of the intact substrate (Figure 3). The fluorescence intensity of the supernatant is measured using a fluorometer and is proportional to protease activity (Mateos et al. 2015).

The FITC‐casein assay is generally considered more sensitive than colorimetric methods like azocasein. In UHT milk shelf‐life studies, FITC‐casein showed the strongest correlations with actual proteolysis compared to azocasein. With extended incubation times, the FITC‐casein assay demonstrated significantly higher sensitivity for detecting very low protease levels, making it more suitable for predicting proteolysis progression in stored milk than the azocasein assay (Button et al. 2011). However, it may still lack the sensitivity required to detect the extremely low protease levels that can cause spoilage over an extended UHT milk shelf life. It is also nonspecific for AprX, detecting plasmin and other proteases active on casein, and requires a separation step (precipitation/centrifugation) and specialized equipment (fluorometer). Assay times can still be relatively long (e.g., a 2‐h assay mentioned as a practical minimum), and potential interference from quenching effects or autofluorescence in the complex milk matrix needs to be considered.

### 2.3 Biosensors

Bioluminescence resonance energy transfer (BRET) assays represent a more recent and sophisticated approach utilizing engineered protein biosensors. A fusion protein is constructed containing a bioluminescent donor enzyme (e.g., Renilla luciferase, RLuc2) and a fluorescent acceptor protein (e.g., green fluorescent protein, GFP2), connected by a specific peptide linker sequence. This linker is designed to be preferentially cleaved by the target protease—in this case, AprX from *P. fluorescens*. When the luciferase substrate (e.g., coelenterazine) is added, the luciferase emits light. If the biosensor is intact, this light energy can be nonradiatively transferred to the nearby fluorescent protein (BRET), causing it to emit fluorescence at its characteristic wavelength. If AprX cleaves the peptide linker, the donor and acceptor are separated, disrupting energy transfer and causing a decrease in the BRET ratio (acceptor emission/donor emission) (Dacres et al. 2023)

This BRET‐based approach has demonstrated significant advantages (Dacres et al. 2023). It is reported to be highly sensitive, with detection limits in the picomolar range (LOD ∼40 pg/mL or 0.8 pM AprX in buffer, ∼100 pg/mL or 2 pM in 50% milk). Crucially, it exhibits high selectivity for *Pseudomonas* AprX activity, showing minimal cross‐reactivity with other proteases tested, including the highly relevant interfering protease, plasmin. The assay is also very rapid, with measurements possible within 10 min, and it has been calibrated using standard enzyme activity units (µU/mL) based on azocasein hydrolysis, facilitating comparisons. Its sensitivity was reported to be approximately 800 times higher than that of the FITC‐casein method in a comparative assay. These characteristics make it a promising candidate both for research and potentially for rapid quality control applications in the dairy industry. However, as a relatively new technology, it requires specialized reagents (the biosensor protein itself) and instrumentation capable of measuring BRET signals (e.g., a filter‐based luminometer or plate reader). Further validation across a wider range of AprX variants from different *Pseudomonas* species and diverse milk matrices may be needed. The cost‐effectiveness for routine industrial use also needs consideration. The development of this BRET technology signifies a potential paradigm shift, directly addressing the critical limitations of sensitivity and specificity that plagued traditional enzymatic assays for AprX in milk.

### 2.4 PCR‐Based Assays

PCR‐based methods for AprX detection rely on amplifying DNA sequences specific to the *aprX* gene, enabling the identification of *Pseudomonas* strains with spoilage potential. Unlike immunological assays, which detect the *aprX* protein itself (regardless of whether it is active) using antibodies, or activity‐based assays, which measure total proteolytic activity but cannot distinguish AprX from other proteases, PCR methods identify the genetic potential for AprX production. Conventional PCR approaches, such as those developed by Marchand et al. (2009b) and Machado et al. (2013), targeted the *aprX* gene to indirectly assess spoilage risk but were constrained by relatively high detection limits in raw milk (∼10^7^ CFU/mL) and a primary focus on *P. fluorescens*, overlooking other proteolytic *Pseudomonas* species. However, these methods could not provide quantitative data without additional steps, limiting their suitability for routine quality control.

A major advancement came with the work of Maier et al. (2021), who developed two triplex qPCR assays for the simultaneous detection and quantification of seven prevalent proteolytic *Pseudomonas* species in raw milk. These assays incorporated species‐specific hydrolysis probes for the *aprX* gene alongside a universal rpoB probe to quantify total *Pseudomonas* populations, enabling both targeted and broad‐spectrum assessment. Analytical performance was robust, with coefficients of determination (*R* ^2^) exceeding 0.975 and amplification efficiencies of 85%–97% across singleplex and multiplex formats. Specificity was rigorously validated against genomic DNA from 75 *Pseudomonas* strains (57 species) and 40 non‐*Pseudomonas* species, confirming accurate discrimination without cross‐reactivity. The assays achieved linear detection across ∼10^3^–10^7^ CFU/mL, matching typical raw milk contamination levels (Ternström et al. 1993), and could differentiate species with varying proteolytic capacities—such as the highly proteolytic *P. proteolytica* and *P. lactis* versus the moderately or weakly proteolytic *P. lundensis* and *P. fragi* (Maier et al. 2021; Marchand et al. 2009a). This species‐level resolution offers critical insight into spoilage potential, allowing for rapid, precise, and actionable monitoring in dairy quality control and targeted interventions to mitigate protease‐mediated spoilage. Beyond single‐target assays, a dual‐quadruplex multiplex qPCR has been reported to quantify major psychrotrophs and enzyme‐coding genes in raw milk with practical sensitivity in spiked and field samples, enabling faster spoilage‐risk screening (Wu et al. 2025).

Despite these strengths, several challenges limit PCR/qPCR as standalone tools for AprX monitoring. They detect genetic potential rather than actual enzyme activity or expression and thus cannot distinguish viable from dead cells or free extracellular DNA. Matrix components in raw milk can further inhibit amplification, necessitating rigorous sample preparation and internal controls. qPCR platforms remain relatively costly and require trained personnel, which may constrain adoption in smaller plants or small‐scale industries. Finally, calibration against culture‐based counts and integration with activity or peptidomic data are essential to translate gene copy numbers into meaningful spoilage risk, adding methodological complexity to routine implementation.

### 2.5 Proteomics and Mass Spectrometry (MS)

In contrast to PCR‐based assays, which detect genetic potential rather than actual enzyme presence or activity, proteomics and MS‐based approaches provide direct molecular evidence of AprX‐mediated proteolysis by identifying and characterizing the peptides generated from casein breakdown. The basic principle involves separating peptides (often via liquid chromatography) and detecting them by mass‐to‐charge ratio, with tandem MS (MS/MS) enabling fragmentation and precise mapping of cleavage sites. Matéos et al. (2015) applied MS/MS to casein substrates hydrolyzed by purified AprX, revealing specific cleavage preferences and substrate specificities that compromise casein micelle stability (Datta and Deeth 2003). Stuknytė et al. (2016) extended this with ultra‐performance liquid chromatography (UPLC)–MS/MS to profile 591 peptides released by *P. fluorescens PS19*, identifying resistant peptides as potential biomarkers for AprX activity in dairy systems.

Marker peptide strategies can also translate proteolytic damage into quantifiable MS readouts; for example, LC–HRMS‐derived marker peptides were implemented in an LC–MS/MS workflow to detect and quantify heat‐resistant proteolytic activity in raw milk (Verhegghe et al. 2021). In this method, LC–HRMS was used to identify signature peptides produced by proteolysis, then peptides were quantified by LC–MS/MS as a direct proxy for the extent of heat‐resistant protease damage in raw milk. Recently, peptidomics has also been applied to interpret spoilage trajectories in processed dairy; for instance, UHT milk spoilage driven by proteases from psychrophilic bacteria has been evaluated using peptidomic signatures (Xu et al. 2024). These high‐resolution peptidomic methods offer unmatched specificity and sensitivity compared to immunological or activity‐based assays, as they can unambiguously attribute proteolysis to AprX and even track temporal patterns of breakdown.

Another important advantage of MS‐based peptidomics is its ability to reveal secondary consequences of AprX activity beyond simple cleavage maps. For example, peptide patterns can be linked to functional changes in solubility, micelle dissociation, heat stability, and gelation behavior, offering mechanistic insight into age gelation or texture defects in UHT milk and cheese (T. Zhang et al. 2024). Coupling peptidomics with metagenomics or metaproteomics also opens avenues to associate specific spoilage phenotypes with particular *Pseudomonas* strains and their protease repertoires.

However, their use is constrained by the need for sophisticated instrumentation, highly trained personnel, labor‐intensive sample preparation, and high operating costs (Aebersold and Mann 2016). Consequently, while they are not suitable for rapid, high‐throughput industrial quality control, MS‐based techniques remain indispensable in research contexts for elucidating AprX mechanisms, validating biomarkers, and informing the design of faster, more practical detection tools.

## 3 Comparative Analysis of AprX Detection Methods

The detection and quantification of AprX in bovine milk require methods that balance sensitivity, specificity, speed, cost‐efficiency, ease of use, and industrial applicability. Also, the suitability of each AprX detection method depends strongly on the dairy matrix under investigation and the stage of the processing chain (raw milk intake, postpasteurization, or UHT shelf‐life monitoring). Table 2 presents a comparative analysis of these key parameters across the major detection methods.

**TABLE 2:** Comparative analysis of current detection and quantification methods for metalloprotease AprX in bovine milk.

Method	Sensitivity	Suitable matrix	Specificity	Advantages	Limitations	References
Indirect ELISA	LOD: 21.0 ng/mL; LOQ: 25.7 ng/mL; lowest detectable activity: 500 pkat/mL	Raw milk, pasteurized milk, UHT milk (postprocessing)	High (AprX‐specific antibodies)	High precision (CV 0.2%–0.8%), good recovery (92%–105%), suitable for raw and processed milk, completion in 6–7 h	Requires two‐step sample treatment; antibody binding depends on sequence homology	Volk et al. 2021
Multiplex qPCR	10^3^–10^7^ CFU/mL	Raw milk (microbial spoilage potential)	High (validated on 75 *Pseudomonas* strains, 57 species)	Simultaneous detection of seven species; quantifies total *Pseudomonas*	Detects gene, not enzyme activity; needs specialized equipment	Maier et al. 2021
Conventional PCR	∼10^7^ CFU/mL	Raw milk (microbial spoilage potential)	Moderate to high	Simple molecular detection across sample types	Limited sensitivity in raw milk; often targets only *P. fluorescens*	Marchand et al. 2009a; Machado et al. 2013
Tandem MS/UPLC–MS/MS	Instrument dependent	UHT milk, stored milk, experimental systems	Very high	Identifies AprX‐specific peptides; maps proteolytic patterns	Expensive; complex prep; high expertise required	Matéos et al. 2015; Stuknytė et al. 2016
Azocasein/FITC‐casein	Low for weak activity	Raw milk, pasteurized milk, UHT milk (total proteolysis screening)	Low	Direct activity measurement; simple and low cost	Nonspecific (detects other proteases); prone to false positives	Christen and Marshall 1984; Christen and Senica 1987
Zymography/milk agar	Low	Concentrated milk extracts, culture supernatants	Low	Visualizes multiple proteases, qualitative confirmation	Semiquantitative; time‐consuming	Stuknytė et al. 2016
BRET biosensor	LOD: ∼40 pg/mL (0.8 pM) in buffer; ∼100 pg/mL (2 pM) in 50% milk	Buffer, diluted milk; emerging for raw/UHT milk	High selectivity for *Pseudomonas* AprX; minimal cross‐reactivity	Rapid (<10 min); ∼800x more sensitive than FITC‐casein	Requires bespoke reagents and BRET‐capable instrumentation; further validation in diverse matrices needed	Dacres et al. 2023

### 3.1 Sensitivity and Specificity

Sensitivity is critical for AprX detection, as even low levels can cause spoilage during extended storage. Indirect ELISA systems, such as that by Volk et al. (2021), achieve excellent sensitivity (LOD = 21 ng/mL) and are valuable for detecting AprX before significant proteolysis. Multiplex qPCR assays (Maier et al. 2021) also show strong sensitivity (10^3^–10^7^ CFU/mL), matching typical *Pseudomonas* loads in raw milk, but detect the *aprX* gene rather than the active enzyme, unlike ELISA. MS (LC–MS/MS/HRMS) offers unmatched specificity by identifying unique AprX‐derived peptides (Parente et al. 2020; Verhegghe et al. 2021) but is costly, equipment‐intensive, and slow for routine QC. Traditional enzymatic assays (azocasein, FITC‐casein) are simple but lack sensitivity for low‐level activity (Baglinière et al. 2013). Emerging biosensors, such as the electrochemical system by Dacres et al. (2023), achieve LODs of ∼40 pg/mL in buffer and ∼100 pg/mL in 50% milk, but require further matrix validation. Synthetic fluorogenic peptides tailored to AprX specificity could provide sensitive, real‐time detection, though current data are limited.

Specificity also varies widely. MS provides the highest specificity by directly confirming AprX activity. Well‐designed ELISAs offer high specificity but may vary with antibody cross‐reactivity. Multiplex qPCR (Maier et al. 2021) achieves species‐level specificity through precise primer/probe design. Enzymatic assays are less specific, detecting all proteases including plasmin (Baglinière et al. 2013), which can yield false positives if used alone. The BRET biosensor (Dacres et al. 2023) combines high sensitivity and specificity but depends on synthetic peptide fidelity and can be affected by milk matrix interference, requiring optimization before industrial use.

### 3.2 Speed and Throughput

In industrial dairy processing, rapid detection is critical for timely quality control and product release, enabling early intervention to prevent spoilage losses, particularly during cold chain storage. Among activity‐based methods, the FITC‐casein assay takes ∼100 min under standard conditions (60 min incubation, 30 min TCA precipitation, 10 min centrifugation), making it unsuitable for high‐throughput screening (Cupp‐Enyard 2009; G. Zhang et al. 2025). The azocasein assay is faster, typically requiring 15 min (Paludetti et al. 2020; Bendicho et al. 2002), though extended incubations (30–180 min) are sometimes used to improve sensitivity or monitor proteolysis kinetics (Leite et al. 2021; Secades and Guijarro 1999), reducing industrial practicality (Baglinière et al. 2013).

Molecular approaches such as multiplex qPCR can deliver results in 3–4 h, including DNA extraction and amplification, with the Maier et al. (2021) system combining this speed with high specificity and sensitivity—suitable for semiautomated, large‐scale operations, though requiring skilled personnel and lab infrastructure. Immunological methods like the indirect ELISA of Volk et al. (2021) take ∼6–7 h, acceptable for end‐of‐day QC but too slow for real‐time process monitoring. MS (Parente et al. 2020) remains the most time‐intensive due to lengthy sample preparation, analysis, and data interpretation, making it more appropriate for confirmatory testing or research.

Emerging biosensor platforms, such as BRET systems (Dacres et al. 2023), offer assay times under 10 min with high sensitivity and specificity, holding strong potential for real‐time industrial monitoring, though further validation in complex milk matrices is required before adoption.

### 3.3 Cost‐Efficiency, Technical Expertise, and Application‐Specific Requirements

Enzymatic activity assays are the most cost‐efficient AprX detection methods, requiring minimal equipment and basic laboratory skills, making them accessible to smaller laboratories. In contrast, MS demands substantial capital investment, ongoing maintenance, and the highest technical expertise for both operation and data interpretation. ELISA, qPCR, and biosensor platforms occupy a middle ground, requiring moderate equipment investment and technical skill; qPCR multiplexing, as in Maier et al. (2021), enhances cost‐efficiency by detecting multiple species simultaneously. ELISA protocols, such as Volk et al. (2021), can be readily implemented in QC labs, while advanced qPCR and BRET biosensors (Dacres et al. 2023) require specialized training.

Matrix complexity in raw milk, which consists of high protein, fat, and native enzyme content, can interfere with detection accuracy. The indirect ELISA of Volk et al. (2021) mitigates this via a two‐step sample treatment, improving sensitivity in untreated milk. Maier et al. (2021) demonstrated that multiplex qPCR correlates well with culture‐based *Pseudomonas* enumeration, supporting its microbiological QC role. BRET biosensors detect AprX at low levels in diluted milk (∼50%) but require further optimization for undiluted raw milk due to quenching and background interference (Dacres et al. 2023). Zymography offers high specificity for proteolytic band visualization but is labor‐intensive and semiquantitative. Traditional azocasein and FITC‐casein assays can estimate total proteolysis but cannot distinguish AprX from other proteases like plasmin; FITC‐casein offers slightly better performance in complex matrices due to improved substrate distribution.

In UHT milk, the focus shifts from detecting *Pseudomonas* cells to measuring residual AprX activity, as the enzyme remains proteolytically active despite severe heat treatment. AprX's high thermal stability (*E*_a_ = 96.1 ± 9.3 kJ/mol; *D*_140°C_ = 124 s) compared to that of plasmin (*E*_a_ = 36.3 ± 12.2 kJ/mol; D_140°C_ = 13 s) means that standard UHT may not inactivate it completely (Kroll and Klostermeyer 1984; Stoeckel et al. 2016; Saint Denis et al. 2001). Immunological and activity‐based assays are therefore more relevant than DNA‐based methods in processed milk, as they detect the enzyme itself. Volk et al. (2021) showed that indirect ELISA can detect AprX post‐UHT, making it suitable for QC. Multiplex qPCR, while effective in raw milk, has limited postprocessing value because gene presence may not correlate with active enzyme. BRET biosensors could be applied to processed milk, but validation is lacking. Azocasein and FITC‐casein assays can detect residual activity, though matrix changes post‐heat treatment can affect sensitivity; they remain useful for total proteolysis assessment when paired with controls.

## 4 Challenges and Limitations

### 4.1 Matrix Complexity and Interference

Milk and dairy products are complex colloidal emulsions with a high content of protein, fat, and various enzymes comprising interconnected activators and inhibitors. Calcium ions play a central role by stabilizing the tertiary structure of AprX. Calcium bridges help the enzyme refold correctly after thermal exposure, thereby enhancing its resistance to heat inactivation (Ertan et al. 2015). Moreover, the presence of lipid membranes and micellar casein can offer protective microenvironments that buffer AprX from inactivation during processing, allowing it to persist even after UHT treatment (Datta and Deeth 2003). In terms of microbial triggers, low‐temperature storage and aeration favor the growth of *P. fluorescens*, which upregulates AprX production, particularly during late log and stationary growth phases (Birkeland et al. 1985; Stoeckel et al. 2016).

In contrast, several inhibitory factors counteract AprX activity. Protease inhibitors naturally present in milk, such as α‐2‐macroglobulin, can nonspecifically bind proteases, though they are more effective against endogenous enzymes like plasmin than bacterial proteases like AprX (Datta and Deeth 2003).

These factors make it difficult to directly detect very low concentrations of AprX without some form of sample manipulation. This has been partly addressed with ELISA methods needing a special preparation method to remove casein interference​ (Volk et al. 2021). Casein molecules in milk are organized into micelles composed of aggregates stabilized by colloidal calcium phosphate (Walstra et al. 2005). In the context of AprX recovery from milk, trisodium citrate has been employed as a preliminary treatment step to release soluble proteins, including AprX, into the supernatant. However, a key drawback of this treatment is that the removal of Ca^2+^ by trisodium citrate also partially inhibits AprX activity, as the enzyme relies on calcium ions for its structural stability and catalytic function (C. Zhang et al. 2019).

Similarly, PCR can be inhibited by milk components if DNA extraction is not efficient. Biosensors and enzymatic assays must function in a milieu where nonspecific adsorption or light scattering can affect readouts. Developing methods that are robust to the matrix or include simple preparation steps is an ongoing need. This reinforces the need for matrix‐aware sample preparation. For example, standardizing a milk pretreatment (like the citrate method or a filtration step) could be key for reproducibility between labs. Volk et al. (2025) introduced a milk‐specific cleanup workflow that removes bulk milk proteins (acidification to ∼pH 5, brief heat treatment, centrifugation) before concentrating AprX. The clarified supernatant is then enriched using high‐salt ammonium sulfate and hydrophobic interaction chromatography, enabling more sensitive downstream AprX detection in milk.

### 4.2 Sensitivity Versus Practicality Trade‐Off

Achieving ultralow detection limits (sub‐ng/mL) often requires sophisticated techniques (BRET, MS, etc.). Not all dairy Quality Assurance laboratories can afford or operate these. While the cost is coming down and devices are becoming user‐friendly, there is always a balance between having a method sensitive enough to catch the smallest problematic levels and keeping it practical. If a test is too sensitive, it might also trigger alarms for situations that would not actually cause spoilage (what one might call “false positives” in a practical sense, though analytically true). For instance, if a milk has 0.05 ng/mL AprX, a BRET sensor will detect it—but perhaps that level would not gel the milk until after 6 months, beyond its normal shelf life. The dairy must decide what threshold is actionable. Defining actionable thresholds for AprX is a challenge that requires more research correlating enzyme levels to spoilage outcomes (initial work suggests 0.3 ng/mL for 4 months and 1 ng/mL for 3 months), but more extensive validation is needed (Dacres et al. 2023). Setting the cutoff too low may lead to the rejection of acceptable milk, whereas setting it too high increases the risk of allowing spoilage to occur.

### 4.3 Strain Variability and Gene Heterogeneity

As noted, different *Pseudomonas* strains have different proteolytic capacities. Dufour et al. (2008) proposed that the level of *aprX* expression is a primary determinant of the variability observed among *Pseudomonas* strains in terms of their milk spoilage potential. This suggests that differences in *aprX* expression may underlie the strain‐dependent proteolytic profiles affecting dairy quality. However, despite this hypothesis, no study to date has directly investigated the relationship between the level of *aprX* expression and the corresponding proteolytic activity, leaving a critical gap in understanding the functional implications of *aprX* regulation in dairy spoilage. Some carry *aprX* but express it poorly; others hyperproduce it (Maier et al. 2020). There are also strains with other proteases (e.g., a strain might produce a serine protease Prt instead of AprX). A single test targeting AprX may miss spoilage due to a non‐AprX protease. However, AprX is by far the dominant enzyme of concern in psychrotrophs (Aguilera‐Toro et al. 2023a). While AprX remains a primary focus in dairy spoilage research, the genetic and proteolytic diversity among spoilage microorganisms necessitates broader detection strategies. Given the variability in protease genes—such as *aprX*, *prtA*, and *prtB*—a single assay may be insufficient for comprehensive monitoring. This highlights the need for multiplex PCR approaches or antibody‐based assays capable of detecting a wider range of proteases across different bacterial species.

Moreover, proteases from *Pseudomonas* are not the only concern. Heat‐resistant proteases secreted by *Bacillus* strains also contribute significantly to milk spoilage. For instance, Choudhery and Mikolajcik (1970) and Santong et al. (2008) reported the presence of thermostable proteases from *Bacillus* species. Yang et al. (2021) evaluated 55 *Bacillus cereus* strains and identified 25 protease producers, with strain C58 exhibiting both strong proteolytic activity and high thermal stability, retaining activity after heat treatments at 70°C for 30 min and at 100°C for 10 min. The active enzyme was characterized as protease HhoA, with a molecular mass of 43.9 kDa. Similarly, Dutt et al. (2009) identified an indigenous *Bacillus subtilis* strain capable of producing a milk‐clotting protease (MCP) with potential implications for dairy spoilage. More recently, Sun et al. (2025) showed the spoilage potential of a thermostable protease from *B. cereus* 12–1 in fluid milk. Therefore, any improvements in AprX detection and control should be embedded within a broader, multitarget surveillance framework for effective dairy quality management.

### 4.4 Standardization and Reference Materials

For chemical and microbiological tests, the dairy industry often has standard methods (e.g., ISO methods for bacterial counts, official AOAC methods, etc.). For AprX detection, methods are still emerging. There is a lack of standard reference materials (like a reference protease preparation of known activity) to calibrate different assays. This can lead to inconsistent results between studies or labs. For example, one laboratory's definition of a protease unit might differ from that of another study. To truly integrate AprX testing, the community would benefit from standard calibration samples (perhaps purified AprX enzyme that can be added to milk as a spike for verifying assay recovery, akin to how labs use reference toxins or reference microbes). Additionally, validating these methods according to regulatory or international standards is a process that takes time. Some companies may be hesitant to rely on a novel method until it has been ring‐trialed and standardized.

### 4.5 Preventative Versus Reactive Culture

Culturally, at the organizational rather than the microbial level, the dairy industry must transition from a reactive to a preventative mindset when addressing enzyme‐driven spoilage. Historically, quality control practices have centered on microbial counts and end‐product testing, such as assessing whether milk gels after extended storage (e.g., 4 months). In contrast, the concept of detecting spoilage‐associated enzymes, such as AprX, at early stages like milk intake or immediately postprocessing, represents a relatively recent paradigm shift. One of the key challenges in promoting this preventative approach lies in demonstrating both the return on investment (ROI) and the reliability of these enzyme‐based diagnostic tools to industry stakeholders. As with any new technology, resistance or inertia, often encapsulated in the mindset of “we've always done it this way,” can hinder adoption. Therefore, early adopters who successfully implement these strategies and document improved shelf life and quality outcomes will play a crucial role in driving broader industry acceptance and cultural change.

### 4.6 Regulatory Frameworks and Quality Standards

The absence of harmonized international standards for AprX detection and quantification represents a significant barrier to widespread adoption of enzyme‐based quality control in the dairy industry. Unlike well‐established microbial parameters, such as total bacterial count or somatic cell count, which have defined regulatory limits across jurisdictions, heat‐stable protease monitoring remains largely voluntary and manufacturer driven (Baur et al. 2015).

Current regulatory frameworks for raw milk quality focus primarily on microbial enumeration rather than enzymatic activity. The European Union's Regulation (EC) No. 853/2004 (European Commission 2004) establishes hygiene standards for raw milk but does not specify limits for psychrotrophic bacteria or their extracellular enzymes. Similarly, the US Grade “A” Pasteurized Milk Ordinance (PMO) sets maximum bacterial limits (e.g., 100,000 CFU/mL for raw milk used in pasteurization) but lacks specific provisions for heat‐resistant proteases (US Department of Health and Human Services 2017). This regulatory gap exists despite evidence that low levels of AprX (undetectable by standard plate counts post‐processing) can compromise UHT milk stability (Stoeckel et al. 2016; C. Zhang et al. 2019).

In contrast, regulatory frameworks for other heat‐stable enzymes provide potential models for AprX standardization. Alkaline phosphatase testing, mandated globally as a pasteurization efficacy indicator, demonstrates how enzyme‐based assays can be integrated into routine quality control through standardized colorimetric or fluorometric methods (ISO 2013). Similarly, the detection of microbial lipases in raw milk, though not universally regulated, has been incorporated into some premium milk procurement schemes with defined activity thresholds (von Neubeck et al. 2015).

Industry‐driven quality assurance programs have begun to address this gap. Some dairy processors now implement internal specifications for psychrotrophic bacterial counts in raw milk (typically <10^4^ CFU/mL) as a proxy for protease risk. However, the absence of validated reference methods and certified reference materials hampers interlaboratory comparability and regulatory acceptance.

The path toward regulatory integration of AprX detection will likely require (1) collaborative method validation through international standards organizations such as ISO or the International Dairy Federation (IDF), (2) establishment of reference materials traceable to SI units, (3) ring trials demonstrating reproducibility across diverse laboratories, and (4) economic evidence linking detection thresholds to measurable improvements in product quality and shelf life. Recent initiatives by the IDF to develop best practice guidelines for heat‐resistant enzyme management in dairy processing signal growing recognition of this need (Machado et al. 2017). Until such standardization is achieved, method selection will remain guided primarily by individual processor requirements rather than regulatory mandates.

## 5 Future Directions

Several promising directions are emerging for the future development of AprX detection methods.

### 5.1 Synthetic Fluorogenic Peptide Substrates

**FIGURE 4:**
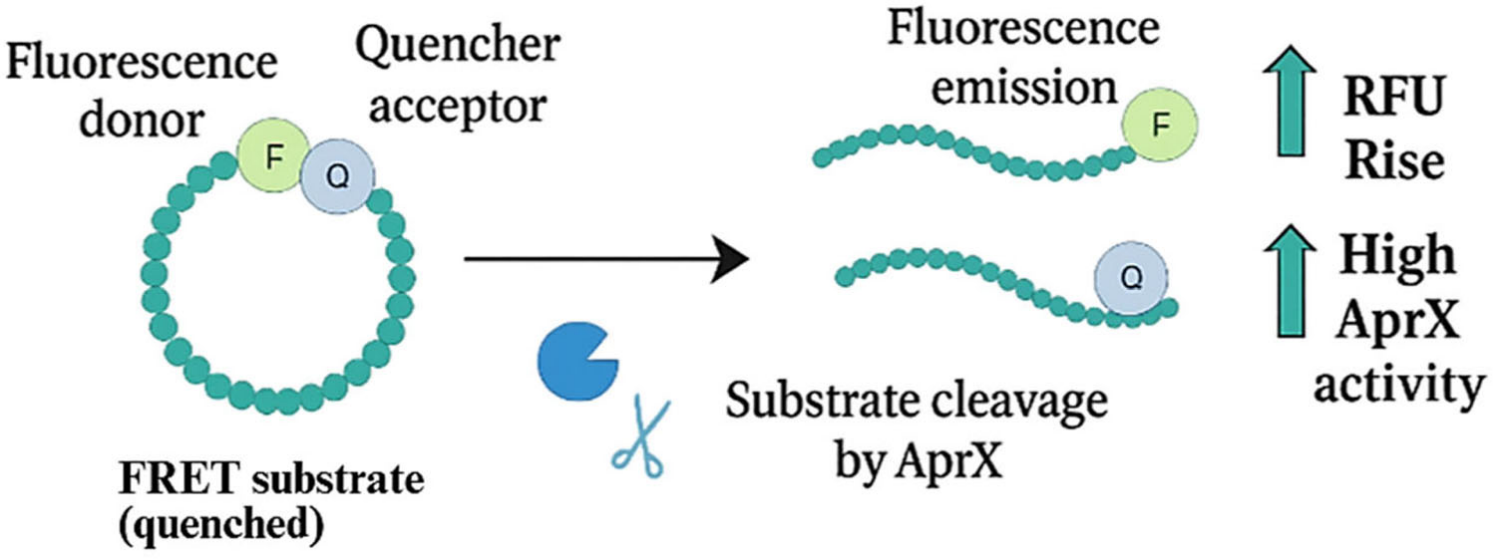
Schematic representation of the FRET‐based assay principle for the detection of AprX protease in milk.

These assays utilize short, custom‐designed peptides linked to fluorescent reporters. A short peptide sequence, designed to mimic a specific protease cleavage site, is synthesized with a fluorophore attached to one end and a quencher molecule to the other. In the intact substrate, the quencher suppresses the fluorescence of the fluorophore through mechanisms like Förster resonance energy transfer (FRET); when a protease cleaves the peptide bond within the linker sequence, the fluorophore and quencher are separated, leading to a measurable increase in fluorescence signal (Figure 4).

These substrates can potentially offer very high sensitivity and allow for continuous, real‐time monitoring of enzyme activity without requiring separation steps. However, their specificity is entirely dependent on the chosen peptide sequence. Designing a peptide sequence that is efficiently and selectively cleaved by AprX but not by plasmin or other interfering proteases in milk is a significant challenge. While promising in principle, their widespread application specifically for AprX detection in milk seems less documented in the materials provided compared to other methods. The cost of synthesizing specific peptides can also be a factor. Furthermore, the activity measured on a short synthetic peptide may not always perfectly reflect the enzyme's activity on complex protein substrates like caseins within the milk environment.

### 5.2 Loop‐Mediated Isothermal Amplification (LAMP)

One significant area of development is the optimization of rapid detection methods, such as LAMP, which have been recognized for their time‐efficient, sensitive, and specific characteristics (Bu et al. 2022; Hu et al. 2022). The application of these methods not only enhances the monitoring of food safety but also supports the simultaneous detection of various components, which can be particularly beneficial in ensuring the quality of dairy products. Further research could focus on the incorporation of these rapid methods into routine quality control processes.

### 5.3 Integration of Multiple Detection Principles

Multiomics approaches—integrating genomics, proteomics, and metabolomics—offer high‐quality tools for the comprehensive characterization of AprX presence, regulation, and activity in dairy systems. Genomic techniques, such as whole‐genome sequencing, can identify the presence of *aprX* and other associated protease genes across diverse *Pseudomonas* strains, while also revealing regulatory elements and mobile genetic elements that may influence their expression (Sirangelo et al. 2023). Emerging tools like CRISPR‐based genome editing further allow functional validation of gene targets, enabling researchers to dissect the genetic determinants that drive AprX production and activity. On the proteomics front, HRMS facilitates precise mapping of casein cleavage products, providing direct evidence of enzymatic activity and substrate specificity. Metabolomics adds another layer by tracking metabolic shifts in milk during spoilage, which can correlate with protease expression and activity profiles. Together, these advanced approaches can deepen our understanding of AprX‐mediated spoilage, uncovering strain‐specific risks and environmental triggers.

### 5.4 Biosensor Development

The development of biosensors incorporating aptamers, nanomaterials, or other specific recognition elements offers the potential for rapid and sensitive detection with simplified instrumentation. One such advancement is a colorimetric biosensor utilizing magnetic nanoparticles (MNPs) designed for the detection of *Pseudomonas aeruginosa* in clinical samples. This platform relies on measuring the proteolytic activity of *P. aeruginosa* using a specific protease substrate (Alhogail et al. 2019). Although not designed specifically for protease detection, a related biosensor was developed for detecting *P. aeruginosa* in whole milk samples. This system featured an antifouling sensing layer based on 3‐(2‐mercaptoethanoxy)propanoic acid (HS‐MEG‐COOH), which was covalently linked to an aptamer for selective binding of *P. aeruginosa* (Spagnolo et al. 2025). Another innovative strategy involves DNAzymes or aptamers that generate a signal upon interaction with a *Pseudomonas*‐specific metabolite or enzyme, although such systems are currently more advanced for medical diagnostics than for detecting the metalloprotease AprX in dairy matrices (Qin et al. 2021). While these approaches primarily target the organism through immunodetection rather than AprX itself, they exemplify the feasibility and utility of rapid pathogen sensing in milk. Collectively, these emerging technologies demonstrate strong potential for point‐of‐use testing and continuous monitoring applications in both clinical and food safety contexts.

### 5.5 Nanoparticle‐Based Assays

Nanoparticle‐based assays provide powerful, miniaturizable platforms for protease detection. Gold nanoparticles (AuNPs) are the most established format to date: peptide‐functionalized AuNPs undergo aggregation or dispersion after proteolysis, giving a red‐to‐blue color change that enables simple, label‐free “mix‐and‐read” assays for serine and cysteine proteases (Chen et al. 2014; Ding et al. 2014).

Quantum dots (QDs) serve as bright, photostable donors in FRET‐type probes, where a protease‐cleavable peptide links QDs to organic dyes or other nanoparticles; cleavage alters energy transfer efficiency, allowing ratiometric or multiplex activity measurements (Kim and Kim 2012). MNPs have been studied in which protease‐sensitive crosslinks between superparamagnetic particles modulate bulk T_2_ relaxation, enabling homogeneous, label‐free detection of cancer‐associated proteases (Zhao et al. 2003). Collectively, these nanoparticle sensors can offer low detection limits, dynamic tunable ranges, and strong potential for point‐of‐care testing. However, true nanoparticle‐based AprX activity assays remain at the prototype stage. Currently, nanoparticles are used more indirectly via recombinase‐aided amplification–lateral‐flow tests targeting the *aprX* gene in *Pseudomonas*‐contaminated milk (G. Zhang et al. 2025).

### 5.6 Online Sensor Integration

Building on the biosensor approach, future dairy plants might incorporate in‐line or at‐line sensors that continuously monitor protease levels during processing. For example, a flow cell could divert a small stream of product through a BRET sensor every few minutes to ensure no protease breakthrough. This would be akin to continuous monitors used for pasteurization (for temperature) or for fat/protein content (infrared sensors). Real‐time monitoring could trigger automatic alarms or diversions if protease activity is detected, preventing a whole batch from being filled into packages. The miniaturization and robustness of sensors will be key here—they must handle high‐temperature UHT environments or be placed post‐UHT in aseptic conditions without contaminating the product. Developments in microfluidics and fiber‐optic biosensors may enable such implementations, providing dairy processors with a “living” insight into enzyme levels at all times.

### 5.7 Artificial Intelligence (AI) and Predictive Analytics

In large‐scale dairy operations, automation of testing is a trend. Robotic samplers and automated analysis (either via flow injection analysis or automated PCR systems) could handle dozens of samples without human intervention. Coupling these automated instruments with data systems and AI algorithms can help predict trends—for example, AI could analyze patterns of AprX detection by farm, season, and so forth to predict when risk is elevated (perhaps warmer months or where certain farm management practices lead to more protease‐producing *Pseudomonas* strains being present). This could proactively inform the supply chain (e.g., flag certain suppliers for deeper cleaning or quicker pickup times). AI could also integrate multiple parameters (plate counts, temperature history, AprX test, plasmin levels, etc.) to give a holistic quality score that is more accurate than any single test.

## 6 Conclusion

AprX, a heat‐stable metalloprotease produced by *Pseudomonas* spp., poses a persistent challenge to the quality and shelf life of dairy products, particularly UHT milk. Its exceptional thermal resistance, broad substrate specificity, and ability to remain active postprocessing demand robust, sensitive, and specific detection strategies. This review synthesizes the current landscape of AprX detection methods, spanning traditional activity‐based assays, immunological approaches, molecular diagnostics, proteomics, and emerging biosensor platforms.

No single method meets all industrial needs. Activity‐based assays remain cost‐effective and simple but lack specificity, while immunoassays such as ELISA offer high sensitivity and matrix adaptability, making them valuable for both raw and processed milk. Molecular techniques, especially multiplex qPCR, excel in species‐level identification and epidemiological tracking but cannot distinguish between active and inactive enzymes. MS and proteomic approaches provide unmatched specificity and mechanistic insight, yet remain impractical for routine use due to cost and technical demands. Emerging biosensors show strong potential for rapid, real‐time detection but require further validation in complex milk matrices.

Future progress will hinge on integrating the sensitivity and specificity of advanced analytical tools with the speed, simplicity, and cost‐effectiveness required for industrial quality control. Hybrid platforms, combining targeted biosensors with confirmatory molecular or proteomic analysis, may offer the most practical route forward. Standardization of protocols and interlaboratory validation will be essential to enable reliable comparisons across studies and to accelerate the translation of promising technologies from research into routine dairy industry practice.

## Author Contributions

**Aritra Sinha**: conceptualization, writing – original draft, data curation, writing – review and editing, investigation, software. **Alan L. Kelly**: supervision, writing – review and editing, funding acquisition, validation, project administration, resources.

## Conflicts of Interest

The authors declare no conflicts of interest.

## Data Availability

No data were used for the research described in the article.
